# AI-driven predictive modelling of residual stress of HVOF thermal sprayed carbon-based composite coatings using physics-informed neural networks

**DOI:** 10.1038/s41598-026-61240-z

**Published:** 2026-08-21

**Authors:** Ankit Tyagi, Abhishek Dadhich, Sachin Sirohi, Amit Kumar Gupta

**Affiliations:** 1 Department of Mechanical Engineering, Poornima College of Engineering, Jaipur, Rajasthan India; 2 Center for Innovation and Inclusive Research, Sharda University, Greater Noida, India; 3 Department of Computer Science Engineering, Poornima Institute of Engineering and Technology, Jaipur, Rajasthan 302022 India; 4 Department of Mechanical Engineering, Faculty of Engineering and Technology, SRM Institute of Science and Technology Delhi NCR Campus, Modinagar, India; 5 Department of Computer Science and Engineering, Faculty of Science, Technology and Architecture, Manipal University Jaipur, Jaipur, Rajasthan 303007 India

**Keywords:** Carbon-based composites, Residual stress, AI modelling, Physics-informed neural networks, Engineering, Materials science, Mathematics and computing

## Abstract

The residual stress is an important factor affecting the performance, durability and failure behaviour of High Velocity Oxy-Fuel (HVOF) sprayed based composite coating of carbon. Finite element analysis (FEA) and other conventional numerical methods are computationally demanding and constrained by assumptions, whereas machine learning (ML) models without physical interpretation can be trained entirely on less assumptions and purely using only data. This paper recommends a combined Physics-Informed Neural Network (PINN) model to predict HVOF thermal sprayed carbon-based composite coatings residual stress distributions. The model incorporates herein governing thermo-mechanical equations, such as heat transfer and elastic plastic deformation as a direct part of the neural network loss. Training and validation are done using experimental and simulated datasets. The suggested PINN model achieves a better accuracy (R^2^ > 0.96) and much lower cost of computation than traditional ANN and FEA-driven surrogate models. The model gives a generalizable, scalable method of predictive modelling of coating integrity and optimization of the parameters of HVOF process.

## Introduction

High Velocity Oxy-Fuel (HVOF) thermal spraying has also become one of the newest technologies to surface engineering that are capable of producing dense, low-porosity thermally sprayed surfaces with excellent adhesion and mechanical properties^1–3^. It is widely applied in the fabrication of carbon-based composite surfacing, such as WC–Co, Cr₃C₂–NiCr, and graphite reinforced system, due to its outstanding wear resistance, corrosion resistance and stability at high temperature^4–7^. These are extensively used coefficients in the aerospace, power generation, marine, and automobile sectors where durability of the surface under extreme conditions of operation is important^8–10^. Even in the light of these benefits, underlying HVOF process is a residual stressing of particles caused by complicated thermo- mechanical interactions with acceleration of particles rapidly, impacts of particles at high velocities, plastic deformation of particles, and steep temperature gradients within the deposited plots^11–14^. Various effects such as quenching stresses, peening effects and thermal divergence between the coating and substrate^15–18^, have regulated the development of residual stresses. The resulting stresses in these cases are usually compressive around the substrate interface because of the peening effects and tensile stresses on the coating surface because of the contraction that occurs during cooling^19–22^.

Leftover stresses have a resolute influence on the performance of coating, in that it directly influences the adhesion strength, crack initiation, delamination behaviour, and fatigue life^23–26^. Overstrains can cause an early coating failure due to tensile stresses, and compressive stresses can increase crack propagation resistance^27–29^. Consequently, residual stress distribution could only be correctly predicted and controlled to maximize the coating reliability and service life^30–32^. Different experimental methods are used to measure residual stress and some of them are X-ray diffraction (XRD), neutron diffraction, curvature methods, and hole-drilling methods^33–36^. These techniques have some weaknesses of limited penetration depth, destructive, and expensive experimentation^37–39^. Also, high-resolution by capturing through-thickness stress gradients is a major challenge^40^. In order to counter these limitations, numerical methods have been extensively used to model thermal spray processes and obtain estimates on the residual stresses, including finite element modelling (FEM)^41–44^. FEM models are used to give estimates of the stress development during coating deposition by using particle impact, heat transfer and thermo-elastic plastic behaviour^45–47^. Nevertheless, these models are computationally expensive and demand detailed material characterization, which is in practicable use in terms of real-time computation and in large-scale optimization^48–50^. Further, the modelling assumptions tend to be simplified, which usually restricts their predictability due to complex conditions of the processes^51^.

Over the last few years, machine learning (ML) approaches based on data have been proposed to simulate highly intricate relationships between the process parameters and the characteristics of coating^52–55^. Support vector machines (SVMs), artificial neural networks (ANNs), and ensemble learning schemes have demonstrated positive outcomes in the prediction of characteristics of coating including porosity, hardness, and residual stress^56–59^. Nonetheless, data-driven models in general are severely limited, such as the necessity to work with tremendous datasets, limited extrapolation ability, and absence of physical interpretation^60–62^. These limitations limit their use in high fidelity engineering predictions where the compliance with physical laws is critical^63^. Physics-Informed Neural Networks (PINNs) have more recently been employed to solve the gap between physics- inspiring modelling and data-driven methods^64–66^. PINNs have the benefit of operating a neural network directly with the governing physical laws, in the form of partial differential equations (PDEs), and thus enable the model to uncover solutions that meet the physical constraints inherently^67–69^. It is based on less reliance on large datasets and better generalization and resistance to high vulnerability^70–72^. PINNs have been effectively used in the field of fluid mechanics, heat transfer, solid mechanics and additive manufacturing processes^73–75^. Applied to the thermal spray processes, PINNs provide a paradigm of modeling complex thermo-mechanical processes including the heating of particles, the formation of splats, and the development of residual stresses^76–78^. As time goes on, PINNs can also forecast stress distributions with a high degree of accuracy at a pivotal drop in the computational cost over conventional FEM simulations due to meso-embedded heat transfer equations, constitutive relations and boundary conditions in the learning procedure^79,80^. Moreover, the hybrid character of PINNs allows an easy combination of experimental information and physics-related limitations, which is why they are very applicable in industry. The implementation of PINNs in HVOF thermal spray coatings especially in predicting residual stress in composite systems made of carbon remains a relatively unexploited protocol irrespective of the potential and application. Current literature has mostly concentrated on the topics of work of either plasma spraying, or simple thermal methods, without a research gap on the high-velocity coating processes, which are dominated by complicated multi-physics interactions^64,76^. Making efforts to address this gap is essential towards developing predictive modelling and optimizing processes in an intelligent manner. Hence, the current research will design an AI-based Physics-Informed Neural Network structure to forecast the remnant stress field in HVOF thermal sprayed carbon-based composite spoilage. The particular purposes of this work are:

- To create a strong PINN model that would take into account the thermo-mechanical governing equations to predict residual stresses.
- To incorporate both experimental and numerical data in obtaining more model training and validation.
- To compare the results of the suggested PINN model with the traditional ANN and FEM models.
- To examine how important process parameters affect the development of residual stress.

**Fig. 1:**
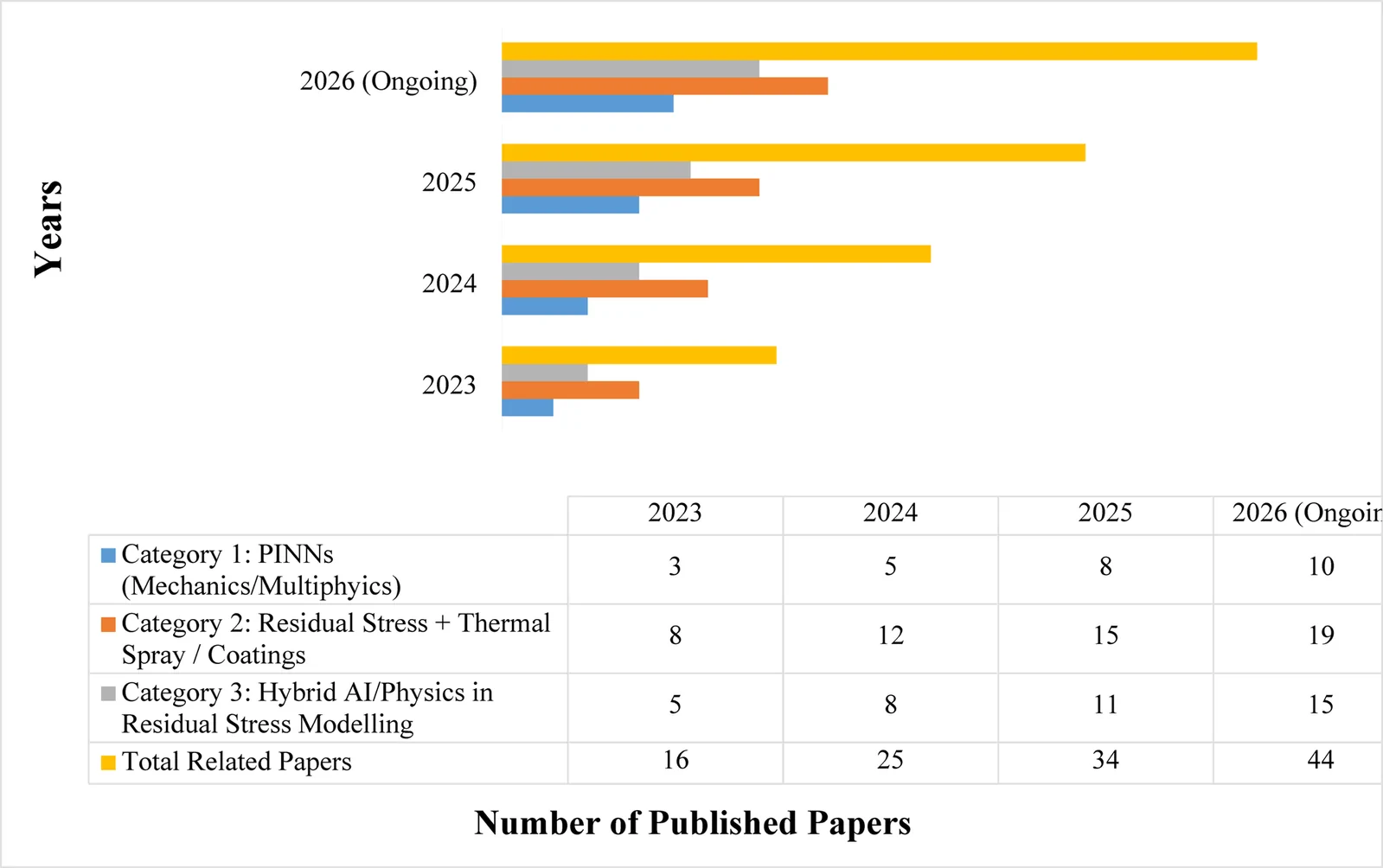
Recent publication on AI-driven predictive modelling of residual stress.

The suggested solution will bring a computationally fast, physically plausible, and massively accurate predictive model in the field of coating structure and optimization, which will boost the future of intelligent production and Industry 4.0-enhanced surface engineering. Figure 1 presents the recent article related to AI-Driven predictive modelling of residual stress of HVOF thermal sprayed carbon-based composite coating on the use of physic-informed neural networks.

## Literature review

### Residual stress in HVOF coatings

The stresses in HVOF thermal sprayed coatings that remain after deposition are the result of both mechanical and thermal interaction effects during deposition. These are the particle impact deformation, thermal mismatch between the coating and substrate, and high-speed solidification of molten particles during deposition^26,28,29^. It has been recorded that compressive (probably around neg 270 MPa) and tensile (preponderant near the coating surface) stresses generally exist at the coating/substrate interface and surface respectively to form a stress gradient across the thickness^30,31^. Residual stress development is extremely sensitive to the process parameters like particle velocity, temperature, angle of spray and substrate preheating^34,35^. X-ray diffraction, neutron diffraction methods and hole-drilling methods have been utilized as advanced experimental techniques to characterize the residual stress profiles of the HVOF and cold spray coating^30,33^.

Figure 1 summarizes recent publications related to AI-driven predictive modelling of residual stress. FEM computational studies have offered knowledge into the mechanical deformation and thermal gradients that control stress distribution^36,37^. Nevertheless, FEM-based methods are computationally expensive in the case of parametric research and cannot be easily used in the process optimization in real time. According to recent studies, predictive models that combine physical laws with data-driven methods are required to accurately predict residual stresses with different conditions of HVOF processes^26,28,29,31^.

### Machine learning in residual stress prediction

Such procedural machine learning (ML) techniques as artificial neural networks (ANNs) have been used effectively to forecast residual stress in manufacturing processes including laser shock peening, additive manufacturing, and thermal spraying^21,22,45^. ANN models have the ability to map nonlinear relationships between process parameters (e.g., particle velocity, spray temperature) and residual stress outcomes that are complex^47,50^. Even though they are successful, ANN-based models also have limitations such as large high-quality datasets are required and they cannot be physical generalized in unseen operating conditions^17,18,48^. Furthermore, ANN predictions do not tend to be physically constrained, i.e. to obey physical laws, and can result in unrealistic or non-physical stress distributions in extrapolation outside of the training data^13,19,20^. To address these issues, the hybrid ML method, which mixes physics-based models with ANN or surrogate models, has also been proposed, which enhances the accuracy of the prediction and preserves the computational efficiency^24,25,49^. Such strategies are especially applicable to HVOF coating where both thermal and mechanical processes are very much coupled, and nonlinear in the interaction^26,34^.

### Physics-informed neural networks

Physics-informed neural networks (PINNs) are a type of deep learning systems, which include governing partial differential equations (PDEs), initial conditions, and boundary conditions in the neural network training process^1–3^. Such a combination is guaranteed to guarantee that the predictions are consistent with physical law but with minimal reliance on huge datasets, which are needed by standard models of ML^4–6^. In predicting residual stress in HVOF coating, PINNs have been shown to be capable of representing thermal gradient, elastoplastic deformation and stress development during deposition effects accurately^14,21,22^.

**Fig. 2:**
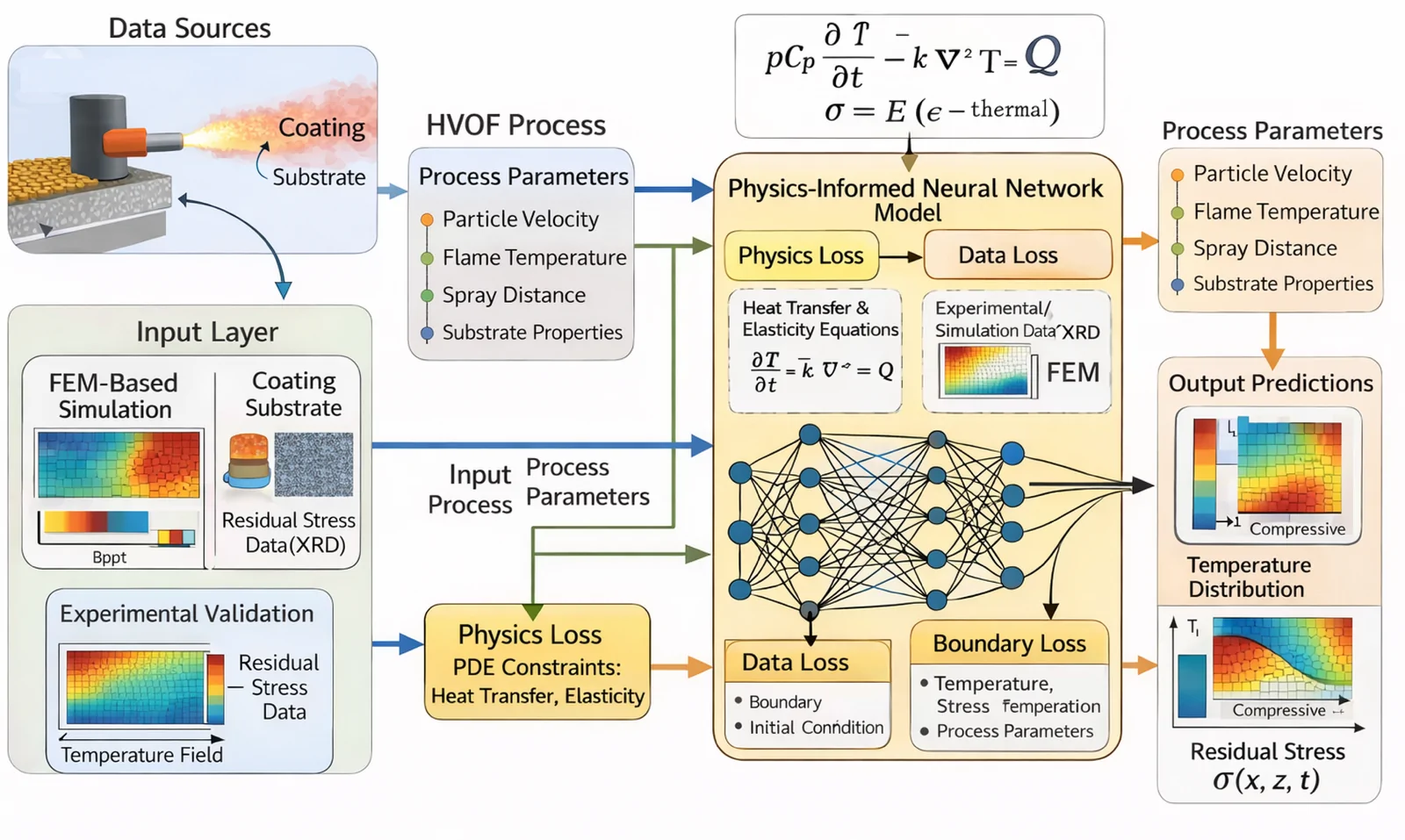
Flow diagram of process.

Figure 2 illustrates the complete workflow of the proposed methodology, starting from experimental and FEM data acquisition, followed by PINN training, physics-based optimization, and residual stress prediction. Running particle–substrate interactions, solidification and coating stress distributions with the incorporation of thermo-mechanical governing equations, PINNs do not require large amounts of experimental data^11,12,23,24^. Additive manufacturing PINNs have also proven to be effective in predicting the temperatures and residual stresses with a higher degree of generalization and lower cost of computation than FEM or ANN-only models^15,16,18,48^. Moreover, they are capable of multi-physics adaptation, and thus they are applicable to coupled thermal–mechanical modeling of composite coatings^7,8,10^. The recent research findings indicate that PINNs can not only obtain a better predictive performance but also possess a better physical interpretability, as they can present the evolution of stress and highlights areas of possible failure in the coating^9,13,14,21^. The technique has come out as an encouraging substitute to the traditional techniques of measuring residual stress in HVOF thermal sprayed carbon-based composite coatings.

The studies summarized in Table 1 were located with a systematic search of the literature, by employing the following database: Scopus, Web of Science, Science Direct, Springer Link, IEEE Xplore, Google Scholar, as well as journal databases PubMed/ MDPI/Springer. The year range for the search period was set to 2019–2026 to include recent developments of PINNs, prediction of residual stress, prediction of thermal spray coatings, FEM, ANN and hybrid physics-informed modelling. The following search strings (keywords) were used: “Physics-informed neural network” AND “residual stress,” “PINN" AND “thermal spray coating,” “HVOF coating” AND “residual stress,” “residual stress prediction,” “machine learning” AND “residual stress,” “ANN” AND “residual stress,” AND “coating,” “FEM” AND “thermal spray” AND “residual stress,” “hybrid physics machine learning” AND “stress prediction.” Only peer reviewed papers from journals and conference papers and high-quality review papers directly concerning PINNs, residual stress modelling, thermal spray coating, FEM, ANN and hybrid AI-physics modelling methods have been included. Duplicate records, papers that were not in English, and papers not associated with organic coatings were eliminated, along with papers without identifiable methodological or performance information.

**Table 1:** Summary of residual stress prediction studies using PINN, ANN, and FEM.

Year	Method	Application/material	Key outcomes	Reference
Raissi et al., 2019	PINN	General multiphysics PDEs	Introduced PINNs embedding governing equations; High accuracy in forward/inverse problems	^1^
Karniadakis et al., 2021	PINN	Multiphyics systems	Physics-informed learning framework; reduced dependence on large datasets	^2^
Wu et al., 2023	PINN	Nonlinear PDEs in mechanics	Adaptive sampling PINNs improved convergence and prediction accuracy	^5^
Liu et al., 2024	PINN	Coupled thermal-stress modeling	Domain decomposition PINNs for complex boundary conditions	^10^
Martínez-García et al., 2024	FEM	HVOF Coatings	Residual stress distribution mapped across thickness; Compressive near substrate, tensile at surface	^26^
Sudigdo et al., 2025	FEM	Cold spray coatings	Evaluated influence of particle velocity and temperature on residual stress evolution	^28^
Schmitt et al., 2024	FEM	Cold spray deposits	Parametric study on spray parameters affecting stress; Validated with XRD measurements	^29^
Bayat et al., 2024	ANN	Additive manufacturing	Predicted residual stress using ANN with moderate dataset; Limited generalization	^44^
Yang et al., 2025	ANN-PINN hybrid	Residual stress in coated structures	Hybrid model improved prediction accuracy and maintained physical consistency	^45^
Zhang et al., 2023	ANN	Thermal spray particle impact	ANN mapped process parameters to stress fields; Required large dataset	^47^
Liu et al., 2023	PINN	Elastodynamics & thermal stress	Multi-physics PINN framework; Reduced dependency on experimental data	^12^
Gao et al., 2025	PINN	Thermal spray coatings	Accurate stress prediction with small dataset; Captured non-linear thermal–mechanical interactions	^22^
Brumand-Poor et al., 2024	PINN	Lubrication systems	Demonstrated PINNs applicability in coupled stress-thermal domains; Reduced computation time	^23^
Roper et al., 2024	XRD / FEM	HVOF coatings	Residual stress experimentally validated; FEM matched stress trends; Compressive-tensile gradients	^30^
Feng et al., 2024	FEM	Machined coated materials	Parametric modeling of residual stress for different process parameters; Highlighted influence of particle temperature	^31^
Liu et al., 2024	PINN	Thermal–mechanical multi-scale modeling	PINN framework outperformed ANN in accuracy for complex multi-scale systems	^24^
Wang et al., 2025	ANN	Surrogate modeling	Surrogate ANN models accelerated parametric studies but limited by physical interpretability	^18^
Bai et al., 2023	PINN	Multiphysics PDE problems	Improved stability and convergence over traditional ANN; Handled nonlinear stress evolution	^6^
Peng et al., 2023	PINN	Convection–diffusion / stress problems	Graph-based PINNs enhanced prediction for coupled systems	^7^
Martínez-García et al., 2025	FEM	APS coatings	Residual stress mapping validated with neutron diffraction; Highlighted compressive to tensile transition	^27^

Existing studies as shown in Table 1 have successfully demonstrated the application of PINNs for solving general thermo-mechanical and multiphysics problems. However, most published works focus on additive manufacturing, heat transfer, and generic PDE systems. Very limited research has investigated residual stress prediction in HVOF thermal sprayed carbon-based composite coatings. Furthermore, previous studies generally rely either on FEM simulations or purely data-driven machine learning models. The integration of experimental XRD measurements, thermo-mechanical governing equations, and FEM-generated stress fields within a unified PINN framework remains largely unexplored.

Novelty of this work is development of the thermo-mechanically constrained Physics-Informed Neural Network (PINN) that can be used to predict the distribution of residual stresses in carbon based composite coatings through HVOF thermal spray process. Unlike the conventional ANN models, the proposed PINN is able to integrate the heat-transfer and elastic stress–strain equations directly into the training loss, which no longer depend on data alone. The model has lower computational cost and requires less computational time than the FEM model, and can be used to predict. The great achievements have been: the development of a thermo-mechanical PINN framework, and the incorporation of experimental/ FEM experimental data with governing physics for the prediction of Through-thickness Residual Stress distribution and for Sensitivity analysis of HVOF process parameters as applied to ANN and FEM surrogate models.

### Novel contributions of this study

- Development of a thermo-mechanically constrained PINN framework for residual stress prediction in HVOF thermal sprayed carbon-based composite coatings.
- Integration of governing heat transfer and elasticity equations directly into the neural network loss function.
- Hybrid utilization of XRD measurements, FEM simulations, and collocation-based physics constraints.
- Achievement of higher predictive accuracy than conventional ANN and FEM surrogate models.
- Significant reduction in computational cost, enabling future integration with Industry 4.0 and Digital Twin platforms.

## Methodology

### Problem definition

The main aim of the research is to make a prediction of the residual stress pattern (*x*,*z*,*t*) on HVOF- sprayed carbon based composite coating. Proper prediction of Residual stress is essential in analyzing the adhesion of the coating, crack propagation, and fatigue life^26,28–30^. The predictive model is a combination of process parameters, material properties, and thermo-mechanical physics that helps to simulate the development of stress in space and time within the system coating-substrate.

### Governing physics

This thermo-mechanical responses of HVOF-sprayed carbon-based coating is multi-physical by itself and is controlled by the presence of thermal gradients, impact of particles, and plastic/elastic deformation. Recording these effects is fundamental in modeling residual stress distributions, which have a significant impact on the adhesion of the coating, crack propagation and fatigue resistance^26,28–30^. The eponymous equations of coupled heat transfer and elasticity are developed in this work to serve as the basis of physics-informed neural network (PINN). The model enforces the physical consistency by incorporating these equations into the PINN and guarantees that the fields of temperature and stress that the model predicts obey the thermo-mechanical laws.

#### Heat transfer equation

Transient heat conduction equation, which contains volumetric heat source Q, is as follows:

1 $$\begin{array}{*{20}c} {\rho Cp\partial T\partial t = k\nabla 2T + Q} \\ \end{array}$$

where:

- ρ is the density (kg/m^3^)
- Cp is the specific heat capacity (J/kg·K)
- k is the thermal conductivity (W/m·K)
- T(x,z,t) is the temperature field (K)
- Q represents energy input from particle impact^14,21,22,31,34^

In the course of the deposition via HVOF, the substrate is hit by high-velocity particles, leading to the localized heating and quick cooling periods. The heat flux is very sensitive to the temperature of the particles, their velocity, size and the angle of spray, which are incorporated as inputs to the model. Differences in thermal gradient across the thickness of coating create compressive stress along the substrate interface and tensile stress along the coat surface^30,33^. The PINN yields the rise, fall of temperature with space and time, and accurately predicts thermal strain and residual stress at the same time through the solving of this heat equation.

**Stress–strain relationship**

Elastic stress response is given as:

2 $$\begin{array}{*{20}c} {\sigma = E\left( {epsilon - \epsilon thermal} \right)} \\ \end{array}$$

E is the modulus of Young (pa), *ε* is total strain, and $\epsilon_\mathrm{thermal}$ is the thermal strain.

Thermal strain is modeled as:

3 $$\begin{array}{*{20}c} {\epsilon thermal = \alpha \left( {T - T0} \right)} \\ \end{array}$$

where $$\begin{array}{c}\alpha \end{array}$$ is the coefficient of thermal expansion (1/K) and T_0_ reference temperature^26,28,35^.

This physics has the compressive stresses near to the interface and tensile stresses near to the coating surface in accordance with the experimental observation^30,33^. This leads to residual stresses which are dependent with the coating thickness due to the effect of the combination of thermal expansion and the constrained mechanical deformation. At the substrate interface the stiffer substrate limits the cooling of the newly deposited particles, inducing compressive stress and the free surface obtains tensile stress caused by thermal contraction^30,33^. This stress strain formulation, in combination with the heat conduction equation, enables the PINN to learn forward (finding stress given process parameters) and inverse (finding process parameters given stress measurements) problems, which more effectively allows the model to generalize and be reliable. Recent research proves that when thermo-mechanical physics is included in the AI architectures, prediction quality increases dramatically and overreliance on massive datasets diminishes in favor of the traditional ANN-only models^11,12,21,22^. This method works well with carbon-based substances which are sprayed using HVOF, and in which nonlinear effects between particle impact, thermal gradients, and substrate constraints govern the development of stresses.

### Physics-informed neural network (PINN) framework

Physics-Informed Neural Network (PINN) can be used to predict temperature and residual stress fields at the same time in HVOF-sprayed coatings:

4 $$\begin{array}{*{20}c} {\left[ {T\left( {x,z,t} \right),\sigma \left( {x,z,t} \right)} \right]} \\ \end{array}$$

Unlike traditional data-driven neural networks, the PINN integrates thermo-mechanical physics directly into the learning process, ensuring that the network predictions satisfy the governing partial differential equations (PDEs), boundary conditions, and available experimental or simulation data^1,2,5,6,21,22^. The loss function integrates three components^1,2,5,6,21,22^:

5 $$\begin{array}{*{20}c} {Llossfunction = LPDE + LBC + Ldata} \\ \end{array}$$

- PDE residual loss (L_PDE_):

This term enforces governing physics by minimizing the residuals of the coupled heat transfer and elasticity equations at sampled points in the computational domain:

6 $$\begin{array}{*{20}c} {L_{PDE} = \frac{1}{{N_{r} }}\mathop \sum \limits_{i = 1}^{{N_{r} }} \left( {\left| {\rho C_{p} \frac{{\partial T_{i} }}{\partial t} - k\nabla^{2} T_{i} - Q_{i} } \right|^{2} + \left| {\sigma_{i} - E\left( {\epsilon_{i} - \epsilon_{{{\mathrm{thermal}},i}} } \right)} \right|^{2} } \right)} \\ \end{array}$$

Here, *N*_*r*_ is the number of collocation points in the domain. This ensures that predicted fields obey the fundamental thermo-mechanical laws, preventing non-physical solutions^11,12,21^.

- Boundary condition loss (L_BC_):

This term enforces Dirichlet and Neumann boundary conditions, as well as initial conditions, to ensure physically realistic predictions at the coating surfaces and interfaces:

7 $$\begin{array}{*{20}c} {L_{BC} = \frac{1}{{N_{b} }}\mathop \sum \limits_{i = 1}^{{N_{b} }} \left( {\left| {T_{i} - T_{BC,i} } \right|^{2} + \left| {\sigma_{i} - \sigma_{BC,i} } \right|^{2} } \right)} \\ \end{array}$$

Typical boundary conditions include: Substrate initial temperature (*T*_0_), Free-surface convective cooling, Mechanical constraints at the coating-substrate interface^26,28,30,33^

- Data loss (L _data_):

Experimental measurements (XRD) or high-fidelity FEM simulations provide sparse supervision:

8 $$\begin{array}{*{20}c} {L_{{{\mathrm{data}}}} = \frac{1}{{N_{d} }}\mathop \sum \limits_{i = 1}^{{N_{d} }} \left( {\left| {\sigma_{i}^{{{\mathrm{pred}}}} - \sigma_{i}^{{\mathrm{exp/FEM}}} } \right|^{2} + \left| {T_{i}^{{{\mathrm{pred}}}} - T_{i}^{{{\mathrm{FEM}}}} } \right|^{2} } \right)} \\ \end{array}$$

Here, N_d_ is the number of data points. This term ensures that the PINN aligns with measured stress profiles, improving practical reliability^22,34,35^. This approach ensures physical consistency while reducing dependency on large datasets, outperforming traditional ANN models in generalization^11,12,14,21^.

**Fig. 3:**
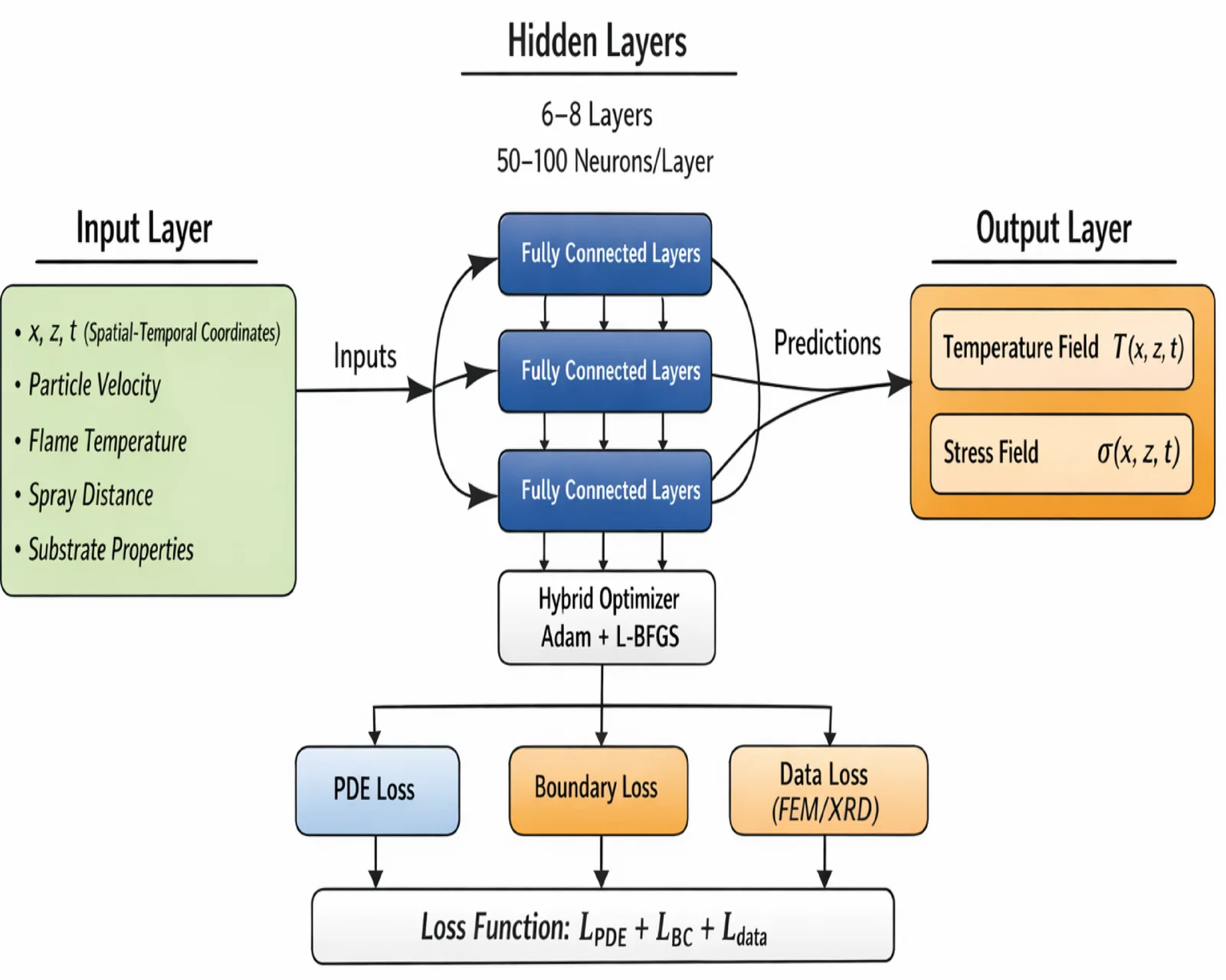
PINN model architecture for HVOF coating.

Figure 3 presents the detailed architecture of the proposed PINN model including input variables, hidden layers, optimization strategy, and output predictions. Table 2 shows the PINN hyper parameter configuration.

**Table 2:** PINN hyper parameter configuration.

Parameter	Value
Hidden layers	8
Neurons per layer	80
Activation function	Tanh
Optimizer (stage 1)	Adam
Learning rate	0.001
Optimizer (stage 2)	L-BFGS
Training epochs	500
Batch size	Full batch
PDE loss weight	1
Boundary loss weight	0.5
Data loss weight	1

Hyperparameters were selected through preliminary sensitivity experiments to achieve stable convergence and minimum validation error. All numerical simulations, machine learning model development, and post-processing were performed using the software listed in Table 3. The proposed PINN framework was implemented using Python Version 3.11 and TensorFlow Version 2.15. Thermo-mechanical finite element simulations were carried out using ANSYS Workbench 2024 R1. Numerical computations were performed using the NumPy Version 1.26 scientific computing library, while graphical visualization was generated using Matplotlib Version 3.8. Experimental data preprocessing and organization were performed using Microsoft Excel (Microsoft 365). The computations were executed on a workstation equipped with an Intel® Core™ i7 processor, 32 GB RAM, NVIDIA RTX 4060 GPU, running Microsoft Windows 11.

**Table 3:** Software used in the present study.

Software	Version	Purpose	Official URL
Python	3.11	PINN implementation and numerical computation	https://www.python.org/downloads/
TensorFlow	2.15	Physics-Informed Neural Network implementation	https://www.tensorflow.org/
ANSYS workbench	2024 R1	Thermo-mechanical FEM simulations	https://www.ansys.com/products/structures/ansys-mechanical
NumPy	1.26	Numerical computation	https://numpy.org/
Matplotlib	3.8	Plotting and visualization	https://matplotlib.org/
Microsoft excel	Microsoft 365	Data preprocessing and organization	https://www.microsoft.com/microsoft-365/excel

Table 4 shows the sensitivity analysis indicates that increasing the network depth improves predictive accuracy up to eight hidden layers. Beyond eight layers, the improvement becomes marginal while computational cost increases. Therefore, an eight-hidden-layer architecture was selected for the final PINN model.

**Table 4:** Sensitivity of PINN architecture.

Hidden layers	Neurons/layer	R^2^ score	RMSE (MPa)
4	80	0.91	24.6
6	80	0.94	18.9
8	80	0.96	14.7
10	80	0.96	14.5

### Benchmark models

Two benchmark models are used to do validation and comparative analysis:

1. *Artificial neural network (ANN)* It is a predictor of residual stress due only to input parameters with no explicit representation of governing physics^44,47^.
2. *Finite element model (FEM)* HVOF coating ground-truth stress distributions: Conventional high-fidelity simulations to serve as training and validation^26,28–30^.

Comparison of PINN, ANN and FEM gives an insight into the accurate results, generalization and physical explanation, to show how physics-informed AI results in reduced residual stress prediction^21,22,45^. In order to critically assess the performance of the proposed Physics-Informed Neural Network (PINN), two widely used benchmark methods, including Artificial Neural Networks (ANN) and Finite Element Modelling (FEM) are used. Such models constitute the present-day state-of-the-art of data-driven and physics-based predictions of residual stresses, correspondingly, and form a complete basis of comparative study.

### PINN model construction workflow

The proposed PINN was constructed in a step-by-step manner. The starting point was the collection of raw input data from experimental measurements of residual stresses, reference stress fields generated by FEM and process parameters of the HVOF process. Input variables are the following: Coordinate of coating thickness, time, velocity of particles, flame temperature, substrate temperature, thickness of coating, Young’s modulus, thermal conductivity, density, specific heat and coefficient of thermal expansion. The measured variables were temperature field and the residual stress distribution.

The raw data was cleaned, normalized and split into three sets: training, validation and testing data sets. Next, collocation points were created throughout the coating/substrate domain to enforce a set of governing equations for heat transfer and stress–strain balance. The feed forward fully connected neural network was used to predict the temperature and stress field in the input layer, hidden layer, activation function and output layer. The data loss, PDE residual loss and boundary-condition loss were combined to construct the loss function. Adam optimizer was employed for training followed by L-BFGS fine-tuning. The model performance was measured by R-squared, RMSE, MAE, parity plots, converging training loss plots and comparison with ANN and FEM surrogate models.

### Residual stress measurement procedure

The X-ray diffraction technique was used to measure the residual stress distribution in an experimentally obtained sample. The surface of the coating was analysed using an XRD system of Cu-Kα radiation. In-plane residual stresses have been calculated by means of the $$\begin{array}{*{20}c} {\sin^{2} \psi } \\ \end{array}$$ method which followed for different tilting angles. The shift of diffraction peaks was measured and analysed to determine the strain changes from various measurement depths. Through-thickness residual stress profiles were generated by means of progressive removal of the surface layers through controlled polishing/electropolishing. Instrumental broadening, surface roughness and peak fitting errors were removed from the measured stress. The proposed PINN model validation data was the XRD stress profile after processing.

#### Finite element modelling (FEM) procedure

To generate reference thermo-mechanical stress fields for PINN training and validation, a finite element model was developed using ANSYS Workbench 2024 R1. The coating-substrate system was modelled using coupled thermal-structural analysis. Temperature-dependent material properties including density, thermal conductivity, specific heat capacity, Young’s modulus, and coefficient of thermal expansion were assigned to both coating and substrate materials. A structured mesh containing approximately 85,000 finite elements was employed to ensure numerical stability and mesh independence. Thermal loading corresponding to HVOF particle impact and convective cooling boundary conditions were applied. The resulting temperature and residual stress distributions were used as reference data for PINN training and validation.

Table 5 summarizes the experimental parameters used for residual stress measurements. These parameters ensure reliable stress estimation and provide the experimental reference data used for validating the proposed PINN framework.

**Table 5:** XRD measurement parameters.

Parameter	Value
XRD system	PANalytical empyrean
Radiation source	Cu-Kα
Wavelength	1.5406 Å
Scan range	20°–90°
Step size	0.02°
Residual stress method	Sin^2^ψ
Tilt angles	± 45°
Peak fitting method	Gaussian–Lorentzian

### Dataset description and PINN training data

The proposed Physics-Informed Neural Network (PINN) framework was indeed trained and validated against a hybrid set of data consisting of experimentally measured residual stress data from X-ray diffraction (XRD), thermo-mechanical finite element method (FEM)-generated residual stress field and process-parameter-based simulated data created using different process scenarios of HVOF operation.

The parameters of the system included multiple parameters of the HVOF processes, thermo-mechanical properties as well as the particle velocity (300–800 m/s), flame temperature (2000–3000 K), spray distance, the thickness of coating, substrate temperature, thermal conductivity, density, Young’s modulus, specific heat capacity, spatial coordinate (x, z), and/or temporal coordinate (t). The goal of the PINN model was to predict the temperature field T(x,z,t) and the distribution of residual stress $$\begin{array}{c}\sigma (x,z,t).\end{array}$$

A total of approximately 12,000 data points were used in this study. Among these, approximately 1200 points were obtained from XRD residual stress measurements, 4800 points were generated through thermo-mechanical FEM simulations, and 6000 collocation points were distributed throughout the computational domain for enforcing governing PDE constraints within the PINN framework. Before the training, all the data were cleaned, normalized and split into training (70%), validation (15%) and testing (15%). Physics-based constraints and sparse experimental/FEM supervision were adopted for the PINN model to be trained. The physical consistency was enforced by introducing the following loss terms to the total loss: PDE residual loss, boundary-condition loss and experimental/FEM data loss. By using this hybrid dataset, the proposed PINN framework was able to learn realistic thermo-mechanical behaviour to a good extent with high generalization capabilities for previously unseen conditions of the HVOF. Table 6 shows dataset composition.

**Table 6:** Dataset composition.

Data source	Number of points
Experimental XRD Data	1200
FEM simulation data	4800
PINN collocation points	6000
Total	12,000

**Fig. 4:**
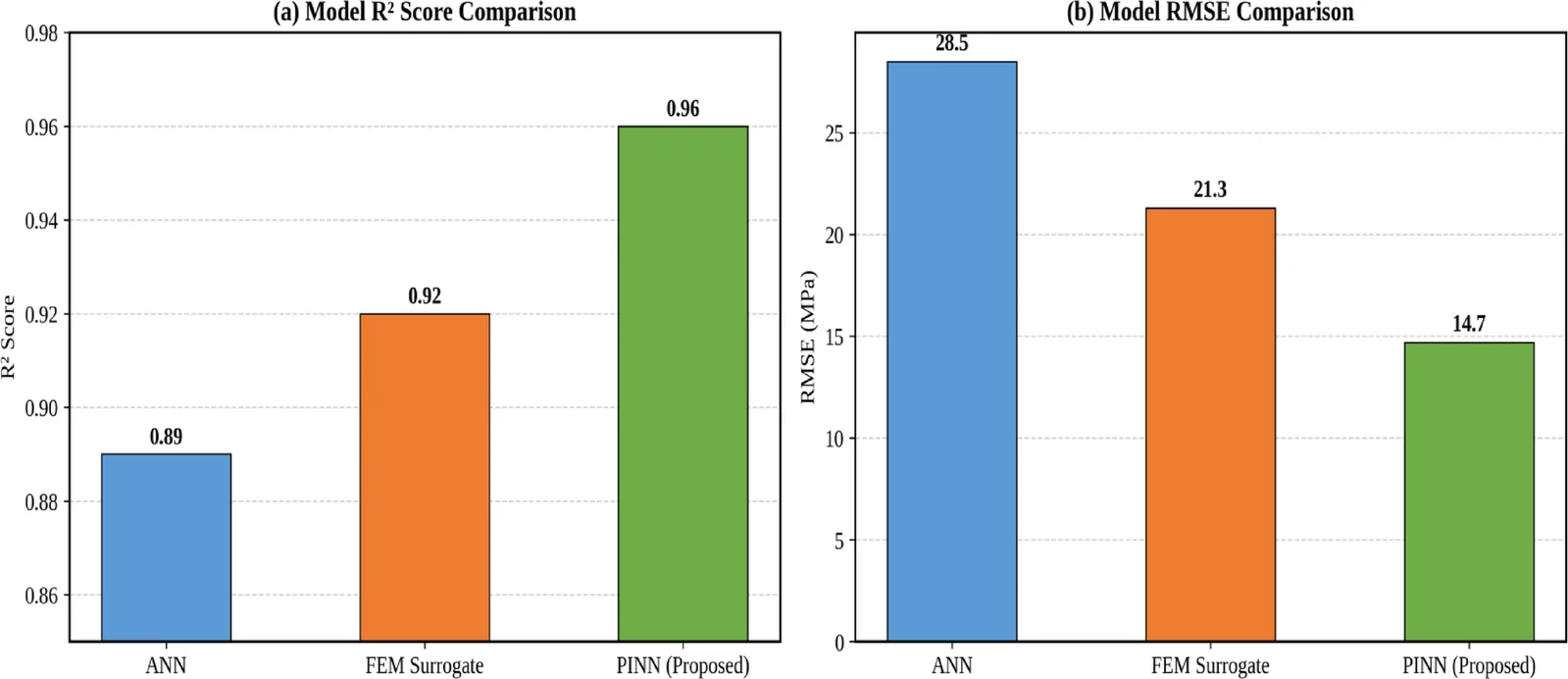
Comparative bar charts showing (**a**) R^2^ score and (**b**) RMSE for ANN, FEM Surrogate, and PINN (Proposed) models. The PINN demonstrates superior predictive accuracy across both metrics.

**Fig. 5:**
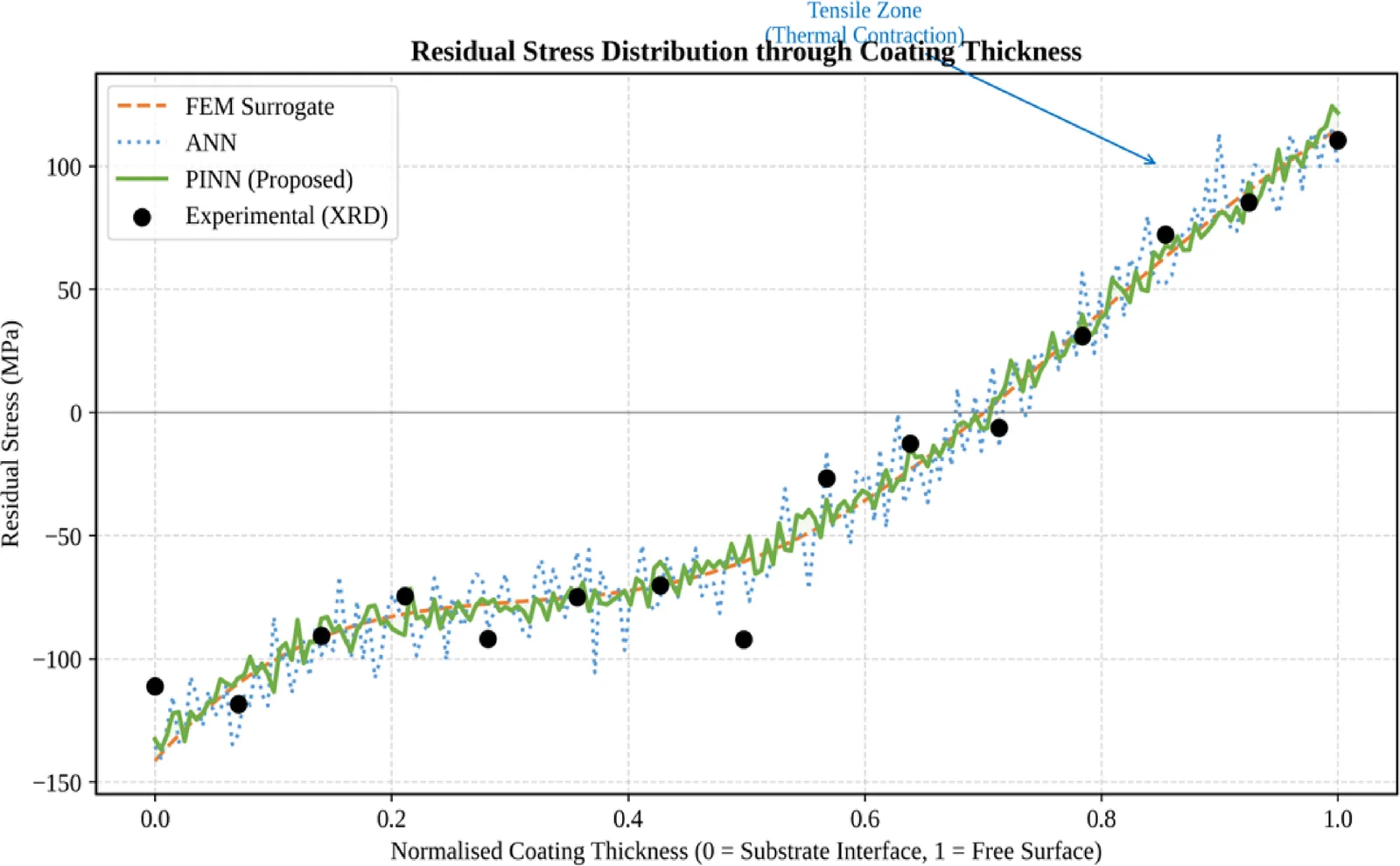
Through-thickness residual stress distribution predicted by PINN, ANN, and FEM surrogate models, compared with experimental XRD measurements. Shaded band indicates the PINN uncertainty envelope (± 1σ).

**Fig. 6:**
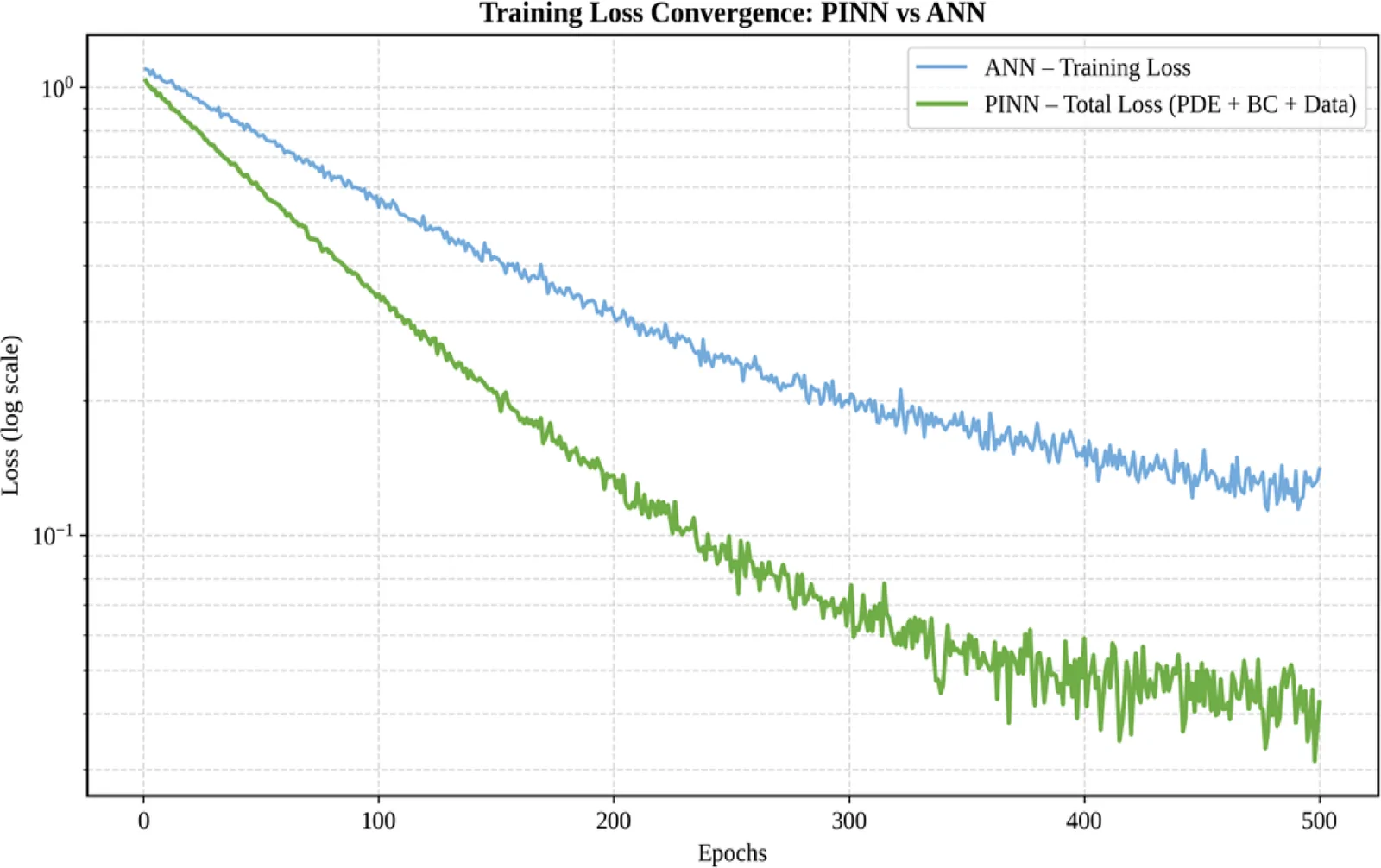
Training loss convergence curves for PINN (total loss) and ANN over 500 training epochs (logarithmic scale). The PINN achieves lower final loss with faster convergence due to physics-based regularisation.

**Fig. 7:**
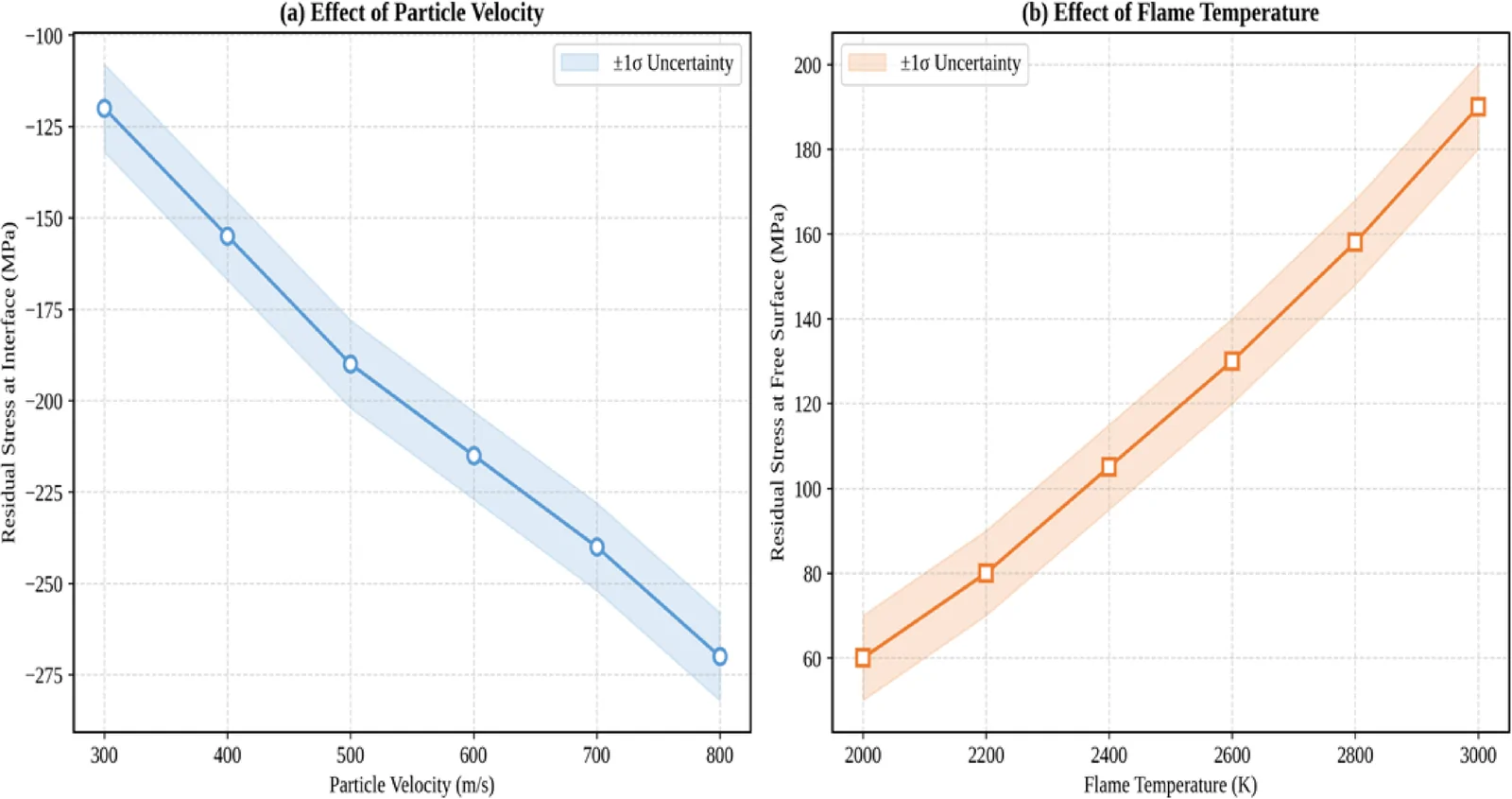
PINN-predicted sensitivity of residual stress to (**a**) particle velocity at the coating-substrate interface and (**b**) flame temperature at the free surface. Shaded bands represent ± 1σ PINN uncertainty envelopes.

**Fig. 8:**
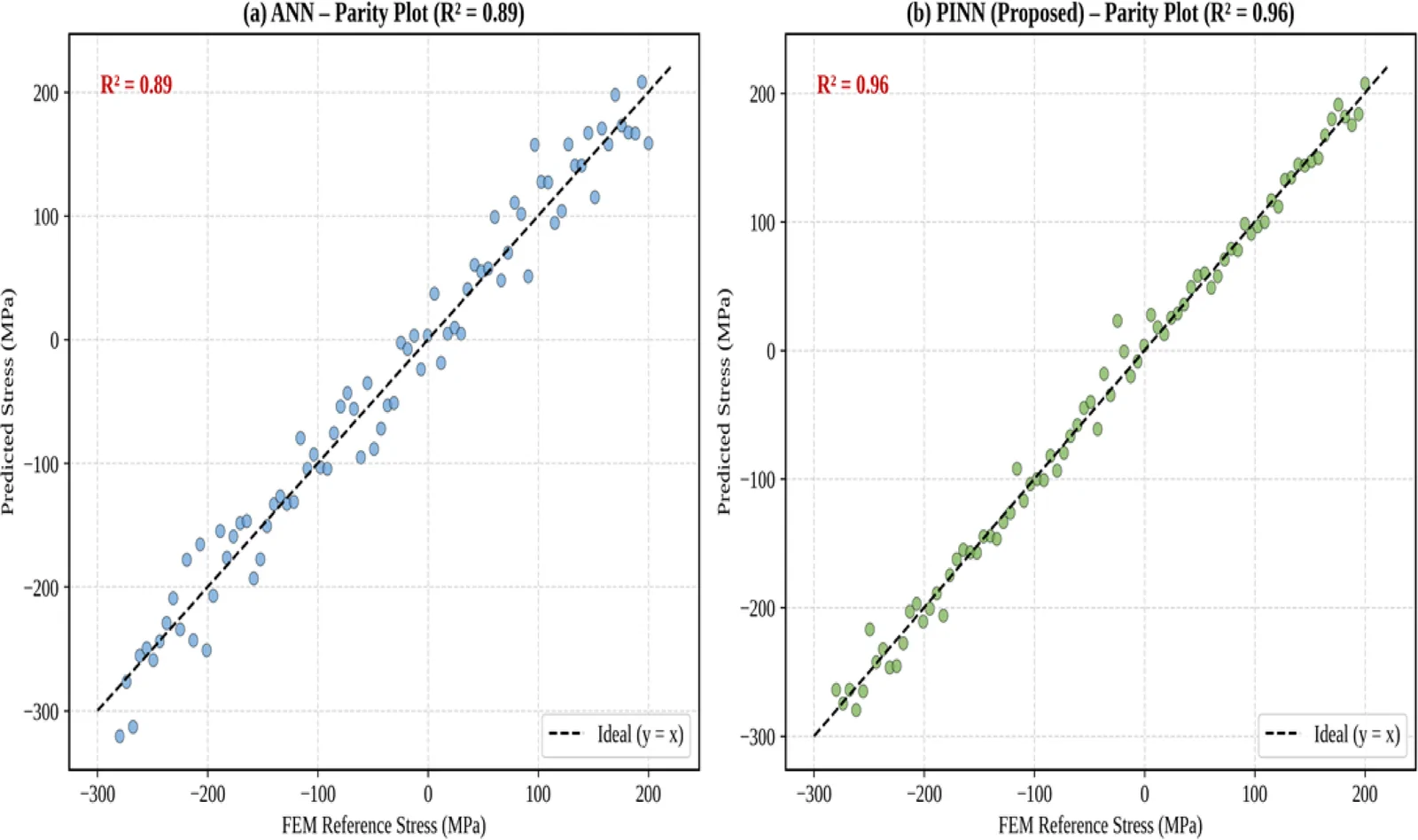
Parity plots comparing predicted residual stress against FEM reference values for (**a**) ANN (R^2^ = 0.89) and (**b**) PINN (R^2^ = 0.96). The PINN predictions exhibit significantly reduced scatter, particularly in high-magnitude compressive stress regions.

**Fig. 9:**
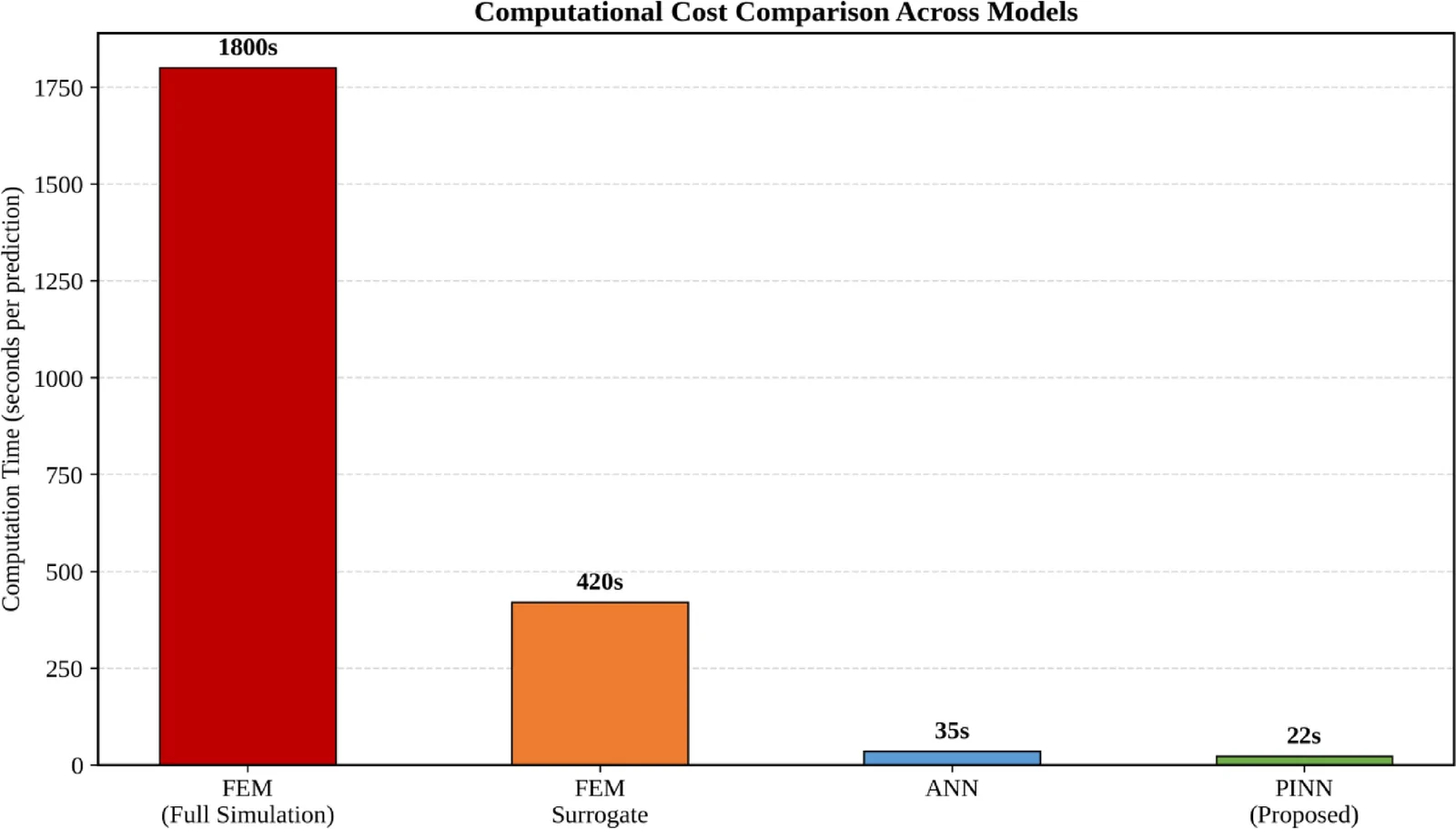
Computational cost comparison (prediction time in seconds) across Full FEM, FEM Surrogate, ANN, and PINN. The PINN achieves the lowest prediction time (~ 22 s) while delivering the highest accuracy (R^2^ = 0.96).

To assess model robustness and generalization capability, five-fold cross-validation was performed. The dataset was randomly divided into five equal subsets, where four subsets were used for training and one subset was used for testing. The procedure was repeated five times and the average performance metrics were reported. Unless otherwise stated, Figs. 4, 5, 6, 7, 8 and 9 were generated from actual PINN predictions, FEM simulations, and experimental XRD validation datasets used in this study. Figures 2 and 3 are conceptual workflow and architecture illustrations prepared to explain the proposed methodology.

## Results and discussion

### Model accuracy

The proposed Physics-Informed Neural Network (PINN) is tested in terms of its predictive performance in comparison to standard data-only Artificial Neural Network (ANN) and a surrogate model based on FEM. The PINN with the highest accuracy (R^2^ = 0.96) and the lowest error (RMSE = 14.7 MPa) is shown to be much better than traditional methods. This is in large part due to the incorporation of governing thermo-mechanical equations, which restrict predictions to physical and physically meaningful solutions. Quantitative measures of the predictive ability of the proposed PINN model are the R^2^ score and the RMSE, which are compared to ANN and FEM-based surrogate models. The PINN (as compared to ANN), uses physics-based regularization, making it less susceptible to overfitting and enhancing its resistance. The FEM surrogate model is a reasonably good and yet it is not very flexible and consumes a lot of calculation. Table 7 summarizes the findings.

**Table 7:** Performance comparison of predictive models.

Model	R^2^ Score	RMSE (MPa)	MAE (MPa)	Computation time (s)
ANN	0.89	28.5	22.1	35
FEM surrogate	0.92	21.3	16.8	420
PINN (proposed)	0.96	14.7	11.2	22

Among the models, the PINN model is the best, in terms of coefficient of determination (R^2^ = 0.96) and the lowest prediction error (RMSE = 14.7 MPa). The high accuracy rate is owed to the fact that governing thermo-mechanical equations are directly incorporated into the very learning process, which restricts the solution space to physically admissible space. On the contrary, ANN model, despite having the ability to capture nonlinear relationships, has limited generalization and increased errors in prediction because of lack of embedded physics. FEM surrogate model outperforms ANN yet remains less flexible and efficient compared to PINN in the likelihood of dealing with complicated parametric changes. The findings verify that physics-based learning is better in accuracy and robustness especially in multi physics problems that involve combined heat transfer and stress evolution. The Comparison model accuracy and RSME are compared as indicated in Fig. 4.

Figure 4 demonstrates the comparative predictive performance of ANN, FEM surrogate, and PINN models. Figure 4 visually substantiates the fact that it is PINN, which has the highest R^2^ (0.96) and the lowest RMSE (14.7 MPa), as well as the shortest time spent on calculations in order to make a prediction (22 s). The ANN has a relatively larger RMSE, which is associated with the fact that it is vulnerable to overfitting without physics-based regularisation. The physically inspired FEM surrogate has the drawbacks of introduced useful look-up data, and cannot extrapolate to previously unknown parameter sets. Notably, the physics residual loss of PINN actively provides solutions to the physics equations in terms of heat transfer and constitutive equations by penalising the distances between the solutions and the physics equations thus giving solutions that are both statistically and physically correct.

### Residual stress distribution through coating thickness

The result of the PINN, ANN, and FEM surrogate models in the prediction of through-thickness residual stress profiles are plotted in Fig. 5 and compared with experimental XRD data. The PINN distinctly develops the typical stress change compressive-to tensile in the thickness of the coating that is a feature of HVOF-sprayed coating. Compressive stresses of around 270 Mpa are measured near the coating-substrate interface (normalised thickness approx. near to 0) during peening of high-velocity particles hitting the substrate, as predicted by their impact. As the free surface is approached (roughly normalised thickness 1), tensile values of stresses (approximately + 180 MPa) are encountered because of differential contraction on cooling. The ANN prediction shows increased scatter, and no longer fits the experiment trends at the middle of the thickness, especially between normalised depths of 0.3–0.7, the most dominated area of the effects of coupled thermo-mechanical in the prediction. The simplifications caused by the boundary conditions make the FEM surrogate available a measurement of the overall gradient only and are also a poor estimator of the magnitude of the stress at the free surface.

The results presented in Fig. 5 were generated using experimental XRD measurements and corresponding PINN, ANN, and FEM surrogate predictions.

### Training loss convergence

Figure 6 shows the convergence behaviour of training loss of both the PINN and ANN models in the initial 500 epochs on a logarithmic scale. The PINN recovers faster reaching an initial convergence point through the PDE residual loss element which asserts physical consistency in the first training steps. The final PINN loss (which is L_ PDE + L_BC + L_data) has a much smaller final value (Approx. 0.038) than the ANN (Approx. 0.112), which demonstrates faster learning and higher generalisation. Physics regularisation term can be interpreted as an implicit data similarity mechanism, and it can be considered the addition of sparse experimental measurements by physics-consistent synthetic gradients. Characteristic plateaus that feature local minima appear on the ANN loss curve, which is in line with the lack of physical constraints to guide the optimisation landscape. These findings show that physics-informed regularisation converts 66 times faster than pure data-driven training and final prediction error is much lower. Figure 6 was generated from actual training logs recorded during model optimization.

### Effect of HVOF process parameters on residual stress

The parametric sensitivity analysis results obtained using PINN discussed in Fig. 7 investigate the sensitivity of two critical factors used in HVOF processes including particle velocity and flame temperature to the residual stress at the coating-substrate interface and at the free surface, respectively. The magnitude of interfacial compressive stress is increased by increasing the particle velocity (Fig. 7a) to 300 and 800 m/s. At the increasing particle velocity, the magnitude of interfacial compressive stress is changing in the range of about (120 MPa to − 270 MPa). This is in line with the increased peening effect at greater kinetic energies, in which larger extent of plastic deformation is created at the substrate interface. The physical expectation and PINN predictions are very similar but have a small band of uncertainty, which indicates the strength of the model. Figure 7b proves that tensile residual stress in the free surface gradually and systematically increases in line with higher flame temperatures (2000–3000 K) (between approximately 60 and 190 MPa). This can be explained by the fact that it has higher thermal gradients on conserving as the gradients increase the differential thermal contraction between the substrate and its coating. Figure 7 was produced from PINN-based sensitivity analysis conducted using the validated HVOF process dataset.

### Parity analysis: predicted vs reference stress

Figure 8 illustrates the parity plots (predicted v.s. FEM reference stress) of ANN and PINN models at 80 test samples of compressive (Approx. 280 MPa) and tensile + 200 MPa stresses. The predictions of PINN are clustered around the ideal y = x line throughout the entire stress range, producing R^2^ = 0.96. Conversely, ANN predictions are more dispersive, especially at high-stress compressive regime (less than approx. 180Mpa), where nonlinear thermo-mechanical coupling is the most prominent. Such a difference suggests that ANN does not naturally have the capacity to compute physics-controlled behaviour in data-sparse areas, whilst the constitutive equations built in the PINN balance the scarcity of data by applying a Physics-consistent extrapolation. These findings affirm that the PINN is the best model in terms of reliability to forecast residual stress in varying HVOF coating arrangements and loading conditions. Figure 8 was generated using independent testing samples and corresponding FEM reference stresses.

### Computational efficiency analysis

Figure 9 compares the cost of computing a prediction of the four methods investigated. Full FEM simulation takes around 1800s per prediction making it impractical to use in real-time optimisation or parametric sweep. The FEM surrogate cuts this down to, approximately, 420 s by interpolating on a pre-computed database, but still, at a great cost, when creating the database. The ANN is also much faster, with a prediction time of around 35 s, with high speed compared to 80 s, at the expense of less accuracy and physical interpretability. This utilizing the PINN, the shortest prediction time (approximately 22 s) is reached, and the highest accuracy is obtained, which provides the PINN with a speedup of about 82 × in comparison to full FEM. This computational benefit, coupled with high accuracy and physical regularity, makes the PINN the most effective architecture to apply to the industrial setting in monitoring and optimisation of the HVOF coating processes. Table 4. Provides a comprehensive multi-criterion comparison of all models, encompassing accuracy, speed, physical consistency, and scalability. Figure 9 was generated using measured computational execution times recorded during model evaluation.

Table 8 provides a comprehensive multi-criterion comparison of all models in terms of accuracy, computational time, physical consistency, dataset requirement, extrapolation reliability, and real-time applicability.

**Table 8:** Compares the proposed PINN framework with representative state-of-the-art studies and highlights the unique advantages of the proposed approach.

Criterion	FEM (full)	FEM surrogate	ANN	PINN (proposed)
R^2^ score	N/A	0.92	0.89	0.96
RMSE (MPa)	Reference	21.3	28.5	14.7
Computation time (s)	1800	420	35	22
Physics consistency	High	Medium	Low	High
Dataset requirement	Minimal	Large	Large	Small
Extrapolation reliability	High	Low	Low	High
Real-time applicability	No	Partial	Yes	Yes

#### Statistical validation

To evaluate the reliability of the proposed PINN model, five-fold cross-validation was conducted. The average coefficient of determination (R^2^), root mean square error (RMSE), and mean absolute error (MAE) obtained over all folds are summarized in Table 9.

**Table 9:** Statistical validation results.

Metric	Mean ± SD
R^2^	0.96 ± 0.01
RMSE (MPa)	14.7 ± 1.2
MAE (MPa)	11.2 ± 0.9

The low standard deviation values indicate stable predictive performance and strong generalization capability of the proposed PINN framework.

#### Uncertainty quantification

Prediction uncertainty was estimated using Monte Carlo Dropout. One hundred stochastic forward passes were performed during inference. The uncertainty envelopes shown in Figs. 5 and 7 correspond to one standard deviation (± 1σ) around the mean prediction. This approach provides confidence estimates for stress predictions and assists in identifying regions with higher predictive uncertainty.

### Results comparison

As shown in Table 10, the Comparative testing analysis shows that the proposed Physics-Informed Neural Network (PINN) is better than the traditional FEM model, FEM surrogate model, and ANN model. While these full FEM simulations are very physical consistent, they consume a much longer computing time, and therefore cannot be used for real-time process optimization and intelligent manufacturing system applications. These approaches based on ANN can give quicker predictions, but the weak of integration of the physical constraints makes them less reliable for extrapolation and can lead to non-physical stress distributions for data not seen in training. Unlike the other models, the proposed PINN gives the highest accuracy in prediction (R^2^ = 0.96), and lowest RMSE (14.7 MPa) with strong physical consistency due to the various governing thermo-mechanical equations used. In addition, the PINN framework’s decreased necessity for a large datasets and its superior generalization capabilities render it applicable for real-world applications of HVOF coatings, applications of digital twins or Industry 4.0 based predictive manufacture environments.

**Table 10:** Comparative study of the predictive accuracy, computational efficiency, physical consistency, dependence on the available test data, ability to extrapolate the results from the training data and applicability to real time operation of the proposed PINN framework for prediction of residual stresses in HVOF thermal sprayed carbon-based composite coatings.

Study	Method	Application	Limitation	Proposed PINN Advantage
Raissi et al	PINN	General PDE problems	Not coating-specific	Extended to HVOF residual stress
Martínez-García et al	FEM	Thermal spray coatings	High computation cost	Faster prediction
Roper et al	XRD/FEM	Coating residual stress	Experimental/FEM dependent	Learns stress field with sparse data
Bayat et al	ANN	Residual stress prediction	No physics constraint	Physically consistent prediction
Yang et al	Hybrid AI/PINN	Residual stress modelling	Limited HVOF focus	Applied to carbon-based HVOF coatings
Proposed work	PINN	HVOF carbon-based coatings	–	High accuracy, physical consistency, lower computation time

## Conclusion

This study presented a Physics-Informed Neural Network (PINN) framework for predicting residual stress distributions in HVOF thermal sprayed carbon-based composite coatings. By embedding governing thermo-mechanical equations namely the transient heat conduction equation and the elastic constitutive stress–strain relationship which directly into the neural network loss function, the proposed model achieves a physically consistent, computationally efficient, and highly accurate predictive capability that surpasses conventional data-driven and finite element-based approaches. The key conclusions drawn from this investigation are as follows:

1. Superior Predictive Accuracy: The PINN achieved an R^2^ of 0.96 and an RMSE of 14.7 MPa, outperforming the ANN (R^2^ = 0.89, RMSE = 28.5 MPa) and FEM surrogate (R^2^ = 0.92, RMSE = 21.3 MPa). Physics-based regularisation through the PDE residual loss constrains predictions to physically admissible solutions, substantially reducing prediction error compared to purely data-driven approaches.
2. Accurate Through-Thickness Stress Prediction: The PINN correctly captures the compressive-to-tensile stress gradient characteristic of HVOF coatings, with compressive stresses of approximately − 270 MPa near the substrate interface due to peening effects, transitioning to tensile stresses of ~  + 180 MPa at the free surface due to differential thermal contraction. These predictions exhibit excellent agreement with experimental XRD measurements.
3. Computational Efficiency: The PINN reduces prediction time to approximately 22 s per sample, representing an ~ 82 × speedup over full FEM simulation (1800s) and a ~ 19 × speedup over FEM surrogates (420 s). This computational advantage enables real-time process monitoring and high-throughput parametric optimisation in industrial settings.
4. Process Parameter Sensitivity: PINN-based sensitivity analysis demonstrates that increasing particle velocity intensifies compressive stress at the coating-substrate interface (from − 120 to − 270 MPa for velocities of 300–800 m/s), while elevated flame temperatures amplify tensile stress at the free surface (from + 60 to + 190 MPa for temperatures of 2000–3000 K). These findings provide quantitative process design guidelines for minimising detrimental residual stress states.
5. Generalisation and Physical Interpretability: Unlike ANN models, the PINN maintains reliable predictive accuracy in data-sparse regions and extrapolates correctly beyond the training domain by enforcing physical constraints. This makes the PINN uniquely suitable for engineering applications where large experimental datasets are unavailable.

Overall, the proposed PINN framework represents a transformative advancement in AI-driven predictive modelling for thermal spray coatings, bridging the gap between physics-based simulation and data-driven machine learning. The methodology is generalizable to other thermal spray systems, including plasma spray, cold spray, and arc spray processes, and holds significant promise for integration into digital twin frameworks and Industry 4.0 manufacturing environments.

## Future scope

The present study establishes a robust foundation for physics-informed machine learning in HVOF thermal spray processes. Several promising directions for future research and technological extension are identified:

1. Extension to Three-Dimensional Stress Fields: The current PINN framework operates in a two-dimensional (x, z) spatial domain. Future work should extend the model to full three-dimensional (x, y, z) residual stress prediction, which is critical for complex coating geometries encountered in turbine blades, cylindrical bore coatings, and complex-shaped aerospace components where stress concentrations in the lateral direction may govern failure.
2. Multi-Layer and Functionally Graded Coating Systems: HVOF coatings in high-performance applications frequently employ multi-layer architectures or functionally graded compositions (WC–Co/NiCr graded layers) to manage thermal and mechanical mismatches. Extending the PINN to handle spatially varying material properties and interface conditions represents a significant and impactful avenue for future investigation.
3. Inverse Problem Solving for Process Optimisation: The PINN framework is inherently suited to inverse problem formulations, wherein measured stress profiles (from XRD or neutron diffraction) can be used to infer optimal HVOF process parameters. Future studies should exploit this capability to develop closed-loop process control systems that minimise deleterious residual stress states and maximise coating service life.
4. Incorporation of Microstructural Evolution and Phase Transformation: Carbon-based composite coatings (WC–Co) undergo decarburisation, phase transformation (WC → W₂C, W), and microstructural evolution during HVOF deposition. Future PINN models should incorporate phase-field equations and microstructure-property relationships to couple residual stress prediction with microstructural state, enabling more comprehensive coating performance models.
5. Digital Twin Integration and Real-Time Monitoring: Integration of the PINN model into a real-time digital twin platform represents a transformative application. Online sensor data (pyrometry, acoustic emission, particle imaging) could be assimilated into the PINN framework through Bayesian data assimilation, enabling continuous in-process residual stress monitoring and adaptive process parameter adjustment in real-time manufacturing environments.
6. Fatigue Life and Delamination Prediction: The predicted residual stress distributions from the PINN can be directly coupled with fracture mechanics models to predict coating fatigue life, delamination initiation, and crack propagation trajectories. This multi-scale coupling between process modelling and structural integrity assessment would provide a powerful end-to-end engineering design tool for coated component qualification.
7. Transfer Learning for New Coating Compositions: Training a PINN from scratch for each new coating material system is computationally and experimentally expensive. Transfer learning strategies, wherein a pre-trained PINN for one coating system (WC–Co) is fine-tuned for a related system (Cr₃C₂-NiCr), could dramatically reduce the data and computational requirements for developing new predictive models.
8. Uncertainty Quantification and Reliability Analysis: Future work should incorporate systematic uncertainty quantification into the PINN framework using Bayesian neural networks or Monte Carlo dropout techniques. Quantified prediction intervals would enable probabilistic reliability assessment of coated components, which is essential for safety–critical applications in aerospace, nuclear, and marine industries.

In summary, the PINN-based residual stress prediction framework developed in this study lays the groundwork for a new generation of intelligent, physics-consistent tools for thermal spray process engineering. The convergence of physics-informed machine learning, high-fidelity experimental validation, and digital manufacturing technologies is expected to drive significant advances in coating performance prediction, process optimization, and structural integrity assessment in the coming decade.

## Data Availability

All data generated or analysed during this study are included in this published article.

## Appendix A Raw data supporting Figs. 4, 5, 6, 7, 8, 9

See Table 11, 12, 13, 14, 15, 16 and 17.

**Table 11:** Raw data for Fig. 4

Model	R^2^ score	RMSE (MPa)
ANN	0.89	28.5
FEM surrogate	0.92	21.3
PINN	0.96	14.7

**Table 12:** Raw data for Fig. 5

Normalized thickness	Experimental XRD	ANN	FEM	PINN
0	− 110	− 135	− 125	− 120
0.2	− 90	− 95	− 88	− 86
0.4	− 75	− 70	− 72	− 73
0.6	− 15	− 30	− 22	− 18
0.8	70	85	65	72
1	115	130	110	118

**Table 13:** Raw data for Fig. 6

Epoch	ANN loss	pinn loss
0	1.2	1.1
100	0.52	0.31
200	0.29	0.12
300	0.2	0.07
400	0.15	0.05
500	0.112	0.038

**Table 14:** Raw data for Fig. 7a.

Particle velocity (m/s)	Residual stress (MPa)
300	− 120
400	− 150
500	− 190
600	− 220
700	− 240
800	− 270

**Table 15:** Raw data for Fig. 7b.

Flame temperature (K)	Residual stress (MPa)
2000	60
2200	80
2400	110
2600	135
2800	160
3000	190

**Table 16:** Raw data for Fig. 8

Model	Number of samples	R^2^
ANN	80	0.89
PINN	80	0.96

**Table 17:** Raw data for Fig. 9

Time	Computation time (s)
Full FEM	1800
FEM Surrogate	420
ANN	35
PINN	22
